# Intravenous Cyclophosphamide Pulse Therapy in the Treatment of Systemic Sclerosis-Related Interstitial Lung Disease: A Long Term Study

**DOI:** 10.2174/1874306400802010039

**Published:** 2008-05-15

**Authors:** C.P Simeón-Aznar, V Fonollosa-Plá, C Tolosa-Vilella, A Selva-O´Callaghan, R Solans-Laqué, E Palliza, X Muñoz, M Vilardell-Tarrés

**Affiliations:** 1 Internal Medicine Department, Vall d´Hebron Hospital, Autonomous University of Barcelona, Bellaterra, Spain; 2 Internal Medicine Department, Parc Taulí Hospital, Autonomous University of Barcelona, Bellaterra, Spain; 3 Radiology Department, Vall d´Hebron Hospital, Autonomous University of Barcelona, Bellaterra, Spain; 4 Pneumonology Department, Vall d´Hebron Hospital, Autonomous University of Barcelona, Bellaterra, Spain

**Keywords:** Systemic sclerosis, interstitial lung disease, cyclophosphamide.

## Abstract

**Objective:**

Interstitial lung disease (ILD) frequently complicates systemic sclerosis (SSc). Cyclophosphamide (CYC) is a promising immunosuppressive therapy for SSc-related ILD. Our objective was to investigate the effectiveness of an intravenous CYC (iv CYC) pulse regime in SSc-related ILD during treatment and thereafter.

**Methods:**

In a prospective observational study ten consecutive patients with SSc-related ILD were treated with iv CYC in a pulse regime lasting from 6 to 24 months. Clinical status, pulmonary functional testing (PFT) and high resolution computed tomography (HRCT) of the chest were evaluated at enrolment and 6, 12 and 24 months thereafter. After treatment withdrawal, patients were followed up every 6 months with PFT and chest HRCT to monitor lung disease.

**Results:**

Clinical improvement was apparent in 8 out of 10 patients. The median values of forced vital capacity (FVC), forced expiratory volume in the first second (FEV1) and diffusion lung capacity for carbon monoxide (DLCO) as well as ground-glass pattern on HRCT did not change significantly after 6, 12 and 24 months of therapy. The follow-up continued in 8 out of 10 patients after treatment withdrawal for a median of 26.5 months (range: 12-48 months). The final median FVC was 54.5% of predicted value (interquartile range, IQR= 31.6%-94%). Only one patient suffered a FVC deterioration greater than 10%, even though less than 160 ml. The final median DLCO was 68% of predicted value (IQR=38.3-83.6%). Only 2 patients who developed pulmonary arterial hypertension deteriorated their DLCO values of more than 15%.

**Conclusions:**

An iv CYC pulse regimen over 24 months may stabilize pulmonary activity in patients with SSc-related ILD during the course of treatment and for a median of 26.5 months thereafter.

## INTRODUCTION

The incidence of pulmonary fibrosis in systemic sclerosis (SSc) varies from 25% to 90%, depending on the method used to identify the interstitial lung disease (ILD) [1]. Pulmonary fibrosis is the major cause of morbidity and mortality in SSc patients [2-6]. Therefore, prompt, accurate diagnosis of lung involvement is necessary in order to prevent the development from the initial alveolitis into the characteristic honeycomb pattern. Disease extent is mainly assessed by pulmonary function testing (PFT) and high resolution computed tomography (HRCT) [7].

While HRCT is the most sensitive and specific tool to identify pulmonary fibrosis in SSc patients, it is not necessarily indicative of the severity of lung impairment [7]. In fact PFT is the only widely available means able to identify whether the disease is sufficiently severe to justify immediate therapeutic intervention.

Several studies support the effectiveness of cyclophosphamide (CYC) in preventing a decline in lung function and delaying death in SSc patients with alveolitis [8-17]. Silver *et al.* [8] first reported that controlling alveolitis with CYC may prevent a deterioration of the pulmonary function. In a large, retrospective cohort study White *et al*. showed that SSc patients who received CYC therapy for pulmonary alveolitis had better lung function outcomes and survival than untreated patients [9]. Recently, two double blind, randomized, placebo-controlled trials have been reported [10, 11]. The first trial, with oral CYC, found a modest but significant beneficial effect on lung function and quality of life, but unfortunately also serious adverse events in six patients [10]. The second trial, with intravenous CYC, demonstrated a non-significant trend towards improved forced vital capacity (FVC) [11]. Thus, both oral and intravenous (iv) CYC therapy have demonstrated their usefulness in previous studies, though the iv route is usually preferred because of its lower toxicity [11-21].

The aim of our study was to prospectively analyze the effectiveness of an iv CYC pulse regimen on SSc-related ILD by PFT and chest HRTC after 6 months, 12 months and 2 years of treatment. A secondary objective was to evaluate the outcome of PFT and chest HRTC after withdrawal of iv CYC therapy.

## MATERIAL AND METHODS

### Population:

The study was performed at the Vall d’Hebron Hospital, a 1,290-bed teaching hospital. SSc patients were recruited from March 2000 to April 2006 at the out-patient clinic for scleroderma Unit of the Internal Medicine Department at our institution. All of them fulfilled the American College of Rheumatology criteria for the diagnosis of SSc [22]. The only criterion for inclusion in the study was chest HRCT compatible with SSc-related ILD and/or FVC ≤ 80% of the predicted value on PFT. Exclusion criteria were: 1) baseline lymphocytopenia of <1,000; 2) any contraindication to CYC use; 3) Cytotoxic drugs or biological response modifying agents for 12 months before entry; or 4) absence of patient’s consent. We evaluated ten consecutive non-smoking SSc patients who had evidence of ILD. The history, duration of lung disease and findings on physical examination were recorded. Patients were classified as having diffuse or limited cutaneous SSc [23].

### Study design:

Single-center interventional prospective study. Patients were treated following the recommendations of Varga J. [24].

### Outcomes:

the primary outcome was to evaluate the effectiveness of the iv CYC pulse regime by PFT and chest HRCT during the treatment and after drug withdrawal. The secondary safety outcome was to estimate the incidence of the side-effects of the drug.

### Protocol for pulmonary evaluation:

PFT and chest HRCT were evaluated at enrolment and after 6, 12 and 24 months of therapy. PFT was performed according to the guidelines of the Spanish Thoracic Society [25, 26] using a Compact MasterLab equipment (Jaeger Healthcare, Hoechberg, Germany).

After discontinuation of the pulse regimen patients were followed up with PFT monitoring every 6 months, for a median period of 26.5 months. PFT included forced spirometry and lung transfer capacity studies (DLCO). Following the American Thoracic Society (ATS) recommendations, these definitions were applied to evaluate changes from entry to final outcome values for FVC, FEV1 and DLCO. Good response to therapy was defined as an improvement in FVC or FEV1 of 10% or more with an increase of at least 200 ml or an improvement of 15% or more in DLCO. Pulmonary function was considered to have worsened if FVC or FEV1 fell by 10% or more with a minimum loss of 200 ml relative to the absolute value, or a decrease in DLCO of 15% or more [27]. Stability was considered when PFT values were between these extremes.

Chest HRCT scan (Philips Tomoscan AV) was performed using a high-resolution protocol of 1.0-1.5 mm width at 15 mm intervals from lung apex to the bases. The sections were acquired in supine position at full inspiration. Two consultant chest radiologists who were unaware of the clinical information scored all scans independently. Each HRCT scan was assessed at 5 levels: origin of the great vessels, middle of the aortic arch, main carina, pulmonary venous confluence and one cm above the right dome of the diaphragm. Each individual section on the HRCT scan was assessed for the presence, pattern, and distribution of interstitial disease. Two distinct patterns of abnormal appearance on the HRCT scan were considered: a ground glass pattern, defined as an increased hazy density of the lung parenchyma, and a reticular pattern, defined as presence of thickened septal or subpleural lines, parenchyma subpleural linear bands, and honeycombing. The findings were defined in accordance with an accepted terminology [28]. The images were scored as follows: at each level the overall extent of reticular pattern and ground glass opacity was visually estimated to the nearest 10% according to MacDonald *et al. *[29]. Overall lung involvement was calculated by averaging the percentages for each zone.

### Treatment:

A new medical circuit was created for this treatment activity, based on the concepts of efficacy, effectiveness and efficiency [30]. Steps for routine administration of iv CYC were as follows: a dose of 0.50-0.75 g/m^2 ^of body surface area was administered in an ambulatory manner to all SSc-related ILD patients enrolled. Ten patients received iv CYC each month for 6 months. In seven patients, the same iv CYC dose was continued bimonthly for 6 months and then quarterly during the second year, as recently recommended [24]. The drug was infused in 500 ml of a 5% glucose solution for 90 minutes. Two separate iv doses of dexametasone 4 mg and ondansetron 4 mg were given to minimize adverse effects. In order to prevent cystitis, iv mesna was given in 3 separate doses and patients were advised to increase their water intake as much as possible, with a minimum of 1,000 cc. All patients were also treated with oral prednisone at a daily initial dose of 50 mg. After one week prednisone was tapered to a daily dose of 5-7.5 mg over 6 weeks, and was then maintained throughout the iv CYC pulse treatment.

### Monitoring of side-effects:

Patients were closely monitored for side effects of the drugs. Karch and Lasagna criteria were used for side-effect diagnosis [31, 32]. Peripheral blood cell count was monitored 15 days after each iv CYC bolus with individual dosage adjustments. Thus, if the total leukocyte cell count was below 3x10^9 ^/ L., iv CYC dose was reduced by 20% in the next pulse treatment.

### Statistical analysis:

Statistical analysis was performed using SPSS (SPSS Inc., Chicago, IL, USA) version 11.0. Measurements were compared between baseline and 6, 12, 24 months using Wilcoxon´s rank sum test.

### Informed consent:

Although this treatment is not recommended in the international guidelines, no alternatives to oral corticosteroids were available during the study period. Therefore, the patients who fulfilled the inclusion criteria specified above were systematically offered treatment with the iv CYC pulse regimen. Treated patients were asked to give written informed consent. Since this is an observational study, the Internal Review Board only required patients to indicate consent by signing their clinical record. For females in fertile status, a report from the gynecologist was required to ensure that pregnancy would not occur.

## RESULTS

Ten patients (9 women and 1 man) were treated with monthly iv CYC pulse. The median age was 49.8 years (range: 23-63 years) and the disease duration previous to treatment was 12.5 years (range: 4-26 years). Seven patients had diffuse SSc and three limited SSc. The median duration of symptomatic lung disease at entry into the study was 7.6 years (range: 3-15 years). None of the patients were active smokers. Clinical and immunological characteristics are shown in Table **1**.

### Clinical evaluation:

All patients received iv CYC pulse each month for 6 months. Three patients discontinued pulse regimen after 6 months, two because of lymphocytopenia before treatment which worsened with its administration, and one patient who died from end-stage ILD-related pulmonary arterial hypertension (PAH). After this initial pulse regimen, seven patients continued bimonthly pulse treatment for 6 months and then completed a second year of treatment.

### Pulmonary Function Testing:

Spirometry results are summarized in Fig. (**1**) and Table **2**. At baseline, seven out of 10 patients had severely reduced FVC (median: 55.5%) and FEV1 (median: 57.3%). Five and three patients respectively presented severe or mild reductions in DLCO. In one patient DLCO could not be determined because of a very low FVC (1.03 litres) and in another DLCO was normal. As a group, the median values for FVC, FEV1 and DLCO did not change significantly after 6, 12 and 24 months of CYC therapy but fulfilled the definition of stable disease. Individually, 7 out of 10 patients showed either improvement or stabilization on PFT after 6 months of therapy. At 12 months, 5 out of 7 patients showed improvement or stabilization. At 24 months, 5 out of 7 patients improved or stabilized.

### Chest HRCT:

Tables **3** and **4** summarize HRCT results. At baseline, all patients showed a ground glass appearance on chest HRCT, and their median percentage was 18%. After 6 months of therapy, the overall ground glass pattern did not change significantly from baseline but individually there was a trend towards a reduction in five out of 10 patients (Table **3**). After 12 months of therapy the ground glass pattern decreased in 2 patients and stabilized in two others, and after 24 months it had fallen in 3 patients and stabilized in one. Before the iv CYC pulse regime a reticular pattern was identified in 7 patients, which did not change significantly after therapy.

### Safety:

The treatment was safe and well tolerated. Only three patients presented decreased white blood cell count after iv CYC boluses and required a dose reduction in subsequent pulses. No patient developed episodes of scleroderma renal crisis, microscopic hematuria, or infection.

### Long-term follow-up after discontinuation of the iv CYC pulse regimen:

Eight out of 10 patients were followed up with clinical assessment and PFT every 6 months for a median of 26.5 months (range: 12-48 months, Table **5**). Two patients were not followed up: one of them died from pulmonary arterial hypertension and the remaining patient just completed the pulse regime. There was a slight but not significant deterioration in DLCO in this treatment-free period. Thus the median DLCO at the end of follow-up was 68% of predicted value, slightly below the median values at baseline (71.7%) and at discontinuation of iv CYC treatment (75%). Interestingly, DLCO deteriorated more than 15% after cessation of iv CYC in only two patients (patients 2 and 4), who developed PAH during this period.

There was no significant change in the median value of FVC at the end of follow-up (54.5%; IQR= 31.6-94%) when compared with the baseline FVC (55.5%; IQR= 32-92.7%) and at cessation of iv CYC (56.7%; IQR= 31.6-82%). Therefore the median deterioration in FVC was only -1.15 % (range: -12.3% to +11.5%).

## DISCUSSION

SSc-related ILD outcome is usually poor when no successful immunosuppressive treatment is administered, and it is associated with increased morbidity and mortality [2, 3, 4]. The optimal treatment for SSc-related ILD patients and the ideal test for evaluating treatment response remain uncertain.

PFT and HRCT were chosen in the present study for the pulmonary evaluation because they are widely considered pivotal in the assessment of ILD. In most studies performed to date lung volumes and DLCO were monitored but results were heterogeneous because of few used the ATS criteria. The notable improvement in DLCO observed by Giacomelli *et al.* [15] was not associated with a significant rise in FVC value, a finding that may reflect the fact that the initial FVC parameter was within the normal range in 17 out of 23 cases. In our study, which applied the ATS criteria, the median values for FVC, FEV1 and DLCO after 6, 12 and 24 months of iv CYC therapy remained stable.

HRCT also has limitations, because the lack of a single validated scoring system to evaluate ILD makes it difficult to quantify the radiological changes reported by different studies. We used HRCT changes as an endpoint because recent data have suggested that it is a good method to determine the morphological extent of alveolitis and fibrosis [29]. Moreover, the literature shows a fairly consistent pattern of positive effects of iv CYC therapy in SSc patients according to HRCT findings: it has been reported that this treatment induces regression of ground glass opacities on HRCT [11, 12, 14], particularly in patients with very early lung disease [16], although sometimes the regression does not reach statistical significance [11, 15, 20]. It should be kept in mind that the small number of patients enrolled in single-center studies might limit the probability of detecting statistically significant improvement in HCRT findings. Taken together, these observations suggest that iv CYC may have a favorable effect on ILD parameters other than PFT.

In our study, there was no statistically significant change in ground glass percentage overall, although individually it fell in 5 out of 10 patients after 6 months of treatment. This probably reflects the favorable response of alveolitis to immunosuppressive therapy, before fibrosis is well established. No changes were observed when a reticular pattern was present.

The most convincing data comes from a recent multicenter, placebo-controlled trial of oral CYC in patients with well-defined, symptomatic SSc-related alveolitis, carried out by the Scleroderma Lung Study Research Group [10]. This report showed modest but statistically significant improvements in lung function and symptoms over one year of follow-up and adds good circumstantial evidence that oral CYC is effective. Unfortunately, the risk of cancer, gonadal failure and other adverse events increases with cumulative dose, especially when the drug is administered daily. Recently, the results of an iv CYC showed a non-significant lung function response to active therapy probably because the sample size was limited, but tolerance and compliance were acceptable [11]. The initial pilot studies [12, 13] and the eight studies published later including a total of 121 patients [14-21] showed consistent evidence of the effectiveness of the iv CYC pulse regimen.

Our prospective study shows that the iv CYC pulse regimen may stabilize ILD activity in patients with SSc both while in treatment and for a median of 26.5 months thereafter. The results of the long-term follow-up were relatively favorable: only two patients experienced a significant deterioration in DLCO, in both cases due to PAH. In the study by Griffiths *et al.* [16], PFT remained stable over 6 months after iv CYC withdrawal but DLCO deteriorated significantly in the majority of patients in further follow-up. The better results obtained in the present study were probably related to the iv CYC pulse regimen. Maintenance pulse therapy for 12 months may achieve further improvement [12, 14]. Some years ago, experts in SSc-related ILD [24] recommended that if a successful clinical and functional response is observed after a six-monthly iv CYC regimen, the interval should be extended bimonthly over 6 months and then quarterly over the second year. To our knowledge, the present study is the only one to have applied this regimen to date and 7 out of 10 of our patients experienced an improvement or stabilization by the end of treatment. Moreover, the absence of any relevant related adverse events demonstrated that this long iv CYC pulse regime was safe.

## CONCLUSION

In conclusion, although our study shares the limitations of other studies in the literature, it suggests that an iv CYC pulse regime over 24 months may stabilize or improve PFT and chest HRCT, with good tolerance in at least some patients with SSc-related ILD. Our findings are consistent with the conclusions of two recently published prospective studies [10, 11].

## Figures and Tables

**Fig. (1).**
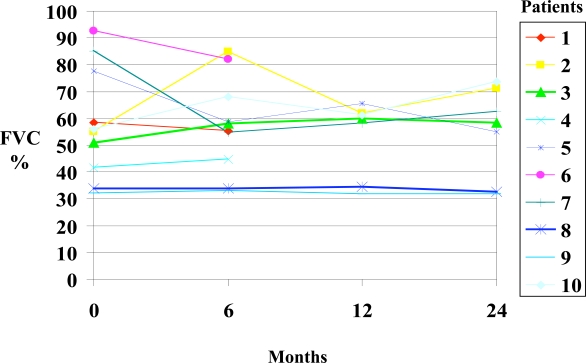
FVC did not change significantly during follow-up.

**Table 1:** Clinical and Demographic Data of SSc Patients

Patient	Gender+	Age (Years)	Disease Duration*(Years)	Cutaneous Subset	Lung Disease++ (Years)	ANA#Title (1:)	Scl-70&	ACA§
1	F	49	15	Diffuse	13	640	Yes	No
2	F	63	11	Limited	4	2560	Yes	No
3	F	44	16	Diffuse	12	320	No	No
4	F	49	26	Limited	8	1280	No	No
5	M	39	4	Limited	4	80	No	No
6	F	59	10	Diffuse	5	1280	Yes	No
7	F	41	8	Diffuse	6	1280	Yes	No
8	F	47	7	Diffuse	6	640	Yes	No
9	F	41	16	Diffuse	15	1280	Yes	No
10	F	23	8	Diffuse	3	160	No	No

+ M: male; F: Female.

* from first SSc symptom to beginning of iv CYC pulse therapy.

++ Duration of lung disease.

# Antinuclear antibodies.

& Scl-70: antitopoisomerase antibodies.

§ ACA: anticentromere antibodies.

**Table 2:** Individual and Median Scores of Pulmonary Function Testing

Patient	FEV1 (%)				FVC (%)			
	Time 0	6 Months	12 Months	24 Months	Time 0	6 Months	12 Months	24 Months
1	56.6	61.8	-	-	58.5	55.4	-	-
2	56.8	84.3	72	73.5	55	84.9	61.7	71.2
3	57.9	57	60	63.7	50.8	58	60	58.5
4	49.4	53.1	-	-	41.6	44.6	-	-
5	89.3	71.7	80.2	69.6	77.7	58.8	65.6	54.9
6	103	90.7	-	-	92.7	82	-	-
7	87.9	66.2	67	71.9	85.2	54.8	58	62.6
8	36	39.7	35.8	36.8	34	34	34.6	32.7
9	41	41.4	42.7	42.7	32	32.8	31.6	31.6
10	68	79.6	72.3	80.4	56	68.1	61.3	73.7
Median	57.3	64	67	69.6	55.5	56.7	60	58.5

**Table 3:** Percentages of Ground Glass Pattern in Serial Pulmonary HRCT

Patients	Baseline (%)	at 6 Months (%)	at 12 Months (%)	at 24 Months (%)
1	32	30	-	-
2	4	6	8	5
3	14	12	14	14
4	32	28	-	-
5	18	28	24	24
6	8	8	-	-
7	26	34	34	30
8	14	14	14	12
9	18	10	10	10
10	32	14	24	10
Median	18	14	14	12

**Table 4:** Percentages of Reticular Pattern in Pulmonary HRCT

Patient	Baseline (%)	6 Months (%)	12 Months (%)	24 Months (%)
1	28	32	-	-
2	0	0	2	0
3	2	2	2	2
4	20	20	-	-
5	0	10	10	10
6	0	0	-	-
7	2	4	2	2
8	8	8	8	8
9	18	22	22	22
10	12	12	14	10
Median	5	9	10	8

**Table 5:** Long Term Results After iv CYC Withdrawal

Patient	Duration of follow Up (Months)	Final DLCO^+(%)^	% Change from DLCO at the End of Treatment	Final FVC^*(%)^	% Change from FVC at the End of Treatment
2	25	68	-24	54.5	-9.8
3	28	72.8	-2.1	54.6	-6.7
4	48	38.3	-44	39.9	-12.3
5	12	52	-13	57	+2.1
6	32	83.6	+1.6	94	+11.5
7	27	60.8	-3.1	66	+1.5
8	26	83	-1	36.8	+10.8
9	14	-	-	31.6	-3.8
Median	26.5	68	-3.1	54.5	-1.15

+ Percentage of predicted DLCO value.

* Percentage of predicted FVC value.
